# Hypoxia Upregulates NOTCH3 Signaling Pathway to Promote Endothelial-Mesenchymal Transition in Pulmonary Artery Endothelial Cells

**DOI:** 10.1155/2021/1525619

**Published:** 2021-11-25

**Authors:** Li-Le Wang, Xiao-Li Zhu, Shu-Hua Han, Lu Xu

**Affiliations:** ^1^Department of Respiratory Medicine, Zhongda Hospital, School of Medicine, Southeast University, Nanjing 210009, China; ^2^School of Medicine, Southeast University, Nanjing 210009, China

## Abstract

**Background:**

To investigate the effect of hypoxia on pulmonary artery endothelial cells and the role of NOTCH3 in endothelial-mesenchymal transition (EnMT) and to provide a research model for pulmonary disease and explain the pathogenesis of the pulmonary disease.

**Methods:**

Pulmonary artery endothelial cells were divided into two groups and cultured in normoxic and hypoxic environments, respectively. QPCR, western blot, and immunofluorescence were used to detect endothelial cell-specific marker protein and mRNA expression in each group, and the ability of endothelial cells migration was evaluated by scratch and transwell experiment.

**Results:**

The pulmonary artery endothelial cells in the normoxic group presented a typical pebble-like arrangement, and the endothelial cells in hypoxic culture showed a long spindle appearance. Hypoxia induced high expression of NOTCH3, Jagged-1, Hes1, c-Src, and CSL. Immunofluorescence showed that endothelial cells in hypoxic culture began to express the *α*-SMA, and the expression of vWF increased with hypoxia. Cell viability, scratch, and transwell results showed that endothelial cells in the hypoxic group were more capable of viability and migration than those in the normoxic group. The induction of EnMT by hypoxia can be inhibited by using notch3-specific inhibitor DAPT and Jagged-1. This study also found that miR-7-5p can regulate endothelial NOTCH3, indicating that miRNA is also involved in the process of endothelial-mesenchymal transformation.

**Conclusion:**

Hypoxia promotes the transformation of endothelial cells into mesenchymal cells by opening the NOTCH3 pathway, which lays the foundation for disease progression or clinical prognosis, and is of great significance in the treatment of diseases.

## 1. Introduction

Pulmonary arterial endothelial cells (PAECs) are monolayer squamous epithelial cells, which are located between the pulmonary circulation blood and the tissues attached to the vessel wall, and are closely attached to the subendothelial tissue through various connective tissues and fibers. Vasodilation and other biological functions: hypoxia participates in various pathophysiological processes in the body, including tissue damage repair, red blood cell generation, tumor development, and tumor growth [1].

The concept of endothelial-mesenchymal transition (EnMT) was proposed in recent years on the basis of epithelial-mesenchymal transition (EMT) [2–5]. As a basic physiological and pathological phenomenon, EMT is widely involved in the formation, development, and invasion of embryos, characterized by the disappearance of skin cell phenotypes and the acquisition of mesenchymal cell phenotypes [6–9]. As a physiological and pathological phenomenon, EnMT is widely involved in embryo formation, development, and neovascularization, during which not only cell phenotypes but also cell markers are changed [10].

During the transformation of epithelial cells into mesenchymal cells, in addition to changes in cell morphology, structure, and cell-to-cell adhesion, the most prominent manifestation is the loss of epithelial-specific marker proteins (such as E-cadherin, Tie1/2, VEGFR, etc.), in turn, obtaining mesenchymal cell-specific proteins, such as Vimentin and fibronectin. The in vivo EMT process is regulated by multiple signaling pathways and acting factors, including the TGF-*β*/Smad signaling pathway [11], PI3K/Akt signaling pathway [12], Wnt signaling pathway [13], Snail and ZEB transcription factors [14–16], and microRNA [17–19]. As a special type of epithelial-mesenchymal transition, endothelial-mesenchymal transition mainly refers to the vascular endothelial cells under certain specific physiological or pathological conditions, the specific antigen (VE-cad, PECAM/CD31) expression gradually disappears, and plasma cell-specific antigens (*α*-SMA and type I and III collagen) begin to express; at the same time, their functional and biological characteristics change significantly, and there is a process of obtaining strong viability and migration capabilities [20–22]. Similarly, EnMT is also involved in the regulation of multiple signaling pathways and signal transduction factors in the body. This study focused on the effects of hypoxia on EnMT. Various studies have proved that the Notch signaling pathway is of great significance in the maintenance of endothelial-mesenchymal transition and the physiological homeostasis of endothelial-mesenchymal transition. This study found that hypoxia promotes mesenchymal transition in endothelial cells by inducing the opening of the NOTCH3 pathway.

The endothelial-mesenchymal transition was found to be involved in embryonic cardiovascular lumen and endocardium formation early. Recent studies have found that there is also a link between EnMT and various diseases. For example, in fibrotic lesions of the heart, lung, and kidney, some activated fibroblasts have been transformed from endothelial cells [23–25]. The cancer-associated fibroblast fraction was also confirmed to be of endothelial cell origin. The activated Notch signaling pathway regulates cell biological events, such as cell proliferation, apoptosis, and survival, and plays an important regulatory role in EnMT. Hypoxia-induced epithelial-mesenchymal transition requires functional Notch signal transduction to drive the EMT process.

As an important signaling system, the Notch signaling pathway is involved in the pathophysiology and inflammatory process of cardiovascular disease. So far, four Notch genes have been found in mammals (Notch 1, 2, 3, 4). The mature Notch receptor molecule is a heterodimer composed of the Notch transmembrane (NTM)/intracellular domain and extracellular Notch (ECN). ECN and NTM are bound together by noncovalent bonds [26]. Notch ligand is a single transmembrane protein that is expressed on the cell surface. The binding of the Notch receptor to the ligand can transmit Notch signals between adjacent cells. So far, Notch ligands have been found to be Jagged-1, Jagged-2, Delta1, Delta3, and Delta4. Cell hypoxia response is not a separate response but intersects with signaling mechanisms, such as Notch signaling. However, the specific mechanism of regulating hypoxic EnMT through the Notch signal transduction pathway is not clear. This study focused on changes in NOTCH3 signaling pathways under hypoxia.

In this study, we observed and identified the process of endothelial-mesenchymal transition induced by hypoxia and explored the possible mechanism of endothelial-mesenchymal transition involved in the occurrence of various diseases.

## 2. Materials and Methods

### 2.1. Cell Culture

Human pulmonary artery endothelial cells (HPAECs) were provided by Guangzhou Geneio Biotechnology Co., Ltd. The cells were cultured in RRPMI 1640 medium containing 10% fetal bovine serum (Gibco, Life Technologies, Rockville, MA, USA), routinely subcultured, and passed to the fourth passage for experiments. [3].

### 2.2. Experimental Grouping

The cells were divided into a normoxic group and a hypoxic group. Normoxic group: the cells are cultured in a normoxic incubator (21% O_2_, 74% N_2_, 5% CO_2_) without other treatment; hypoxia group: cells are placed in a three-gas incubator (1% O_2_, 94% N_2_, 5% CO_2_) culture. In addition, the experimental group also includes DAPT (–) and DAPT (+) groups under hypoxia induction. Si-NC and si-Jagged-1 group, mimics-NC, miR-7-5p mimics, inhibitor-NC, and miR-7-5p inhibitor groups were transfected with Lipofectamine 2000 (Life Technologies, Rockville, MA, USA).

### 2.3. Scratch Test

The third-generation endothelial cells were seeded into a 6-well plate, and after the fusion into a single layer, a straight line was drawn with a sterile pipette compared to a ruler. The pipette head was as perpendicular to the inner surface of the plate as possible, so that the straight line passed through the middle of each well [5]. The cells were washed 3 times with PBS to remove the cut cells, and a serum-free medium was added. 6-well plates were placed in 37°C normoxic chambers and a hypoxia chamber, respectively, and cultured. Take samples and take photos to observe the migration of endothelial cells, and observe the migration distance of endothelial cells in normoxic and hypoxic environments.

### 2.4. MTT Colorimetric Method to Detect Cell Viability

HPAEC cells were collected in the logarithmic growth phase and the cell density was counted and adjusted, and 5 × 103 cells/well were inoculated into 96-well plates and synchronized with the 1640 medium of 0.5% FBS for 24 hours to make the cells in the same growth cycle. The corresponding concentration of reagents was added according to the grouping method. 5 duplicate wells were set in each group and incubation was continued in the incubator. After 72 h, 10 *μ*L (5 g/L) of MTT (Beyotime, Shanghai, China) was added to the medium, the operation was protected from light as much as possible, and the culture was continued in an incubator. [5] After 3 h, the 96-well plate was removed, the culture medium was removed, 150 *μ*L of DMSO (Sigma-Aldrich, St. Louis, MO, USA) was added to each well, it was shaken uniformly, the absorbance A was measured at a wavelength of 570 nm using a microplate reader, and the cell growth inhibition rate was calculated. Cell growth inhibition rate = (1−experimental group A/model group A) × 100%.

### 2.5. Transwell Assay for Cell Migration

HPAEC cells were collected by digestion, and the cell density was counted and adjusted. 5 × 10^5^ cells per well were seeded in 6-well plates. The 1640 medium of 0.5% FBS was synchronized for 24 hours so that the cells were in the same growth cycle. The culture medium was removed, the corresponding concentration of reagents was added according to the grouping method, 2 mL per well was added, and cells were placed in an incubator for cultivation. After 72 hours of incubation, cells were digested and collected. The cells were resuspended and counted in a serum-free medium. The upper chamber (Millipore, Billerica, MA, USA) was inoculated with serum-free cell suspension, and the inoculum per well was 5 × 10^5^. The lower chamber was added with a 10% serum-containing medium. Incubator Medium routine is for 8 h. The upper chamber liquid was blotted dry, rinsed once with PBS, and 400 *μ*L of methanol was added and fixed at room temperature for 10 min. Absorb the upper chamber fixing solution, add 400 *μ*L of crystal violet staining solution, and gently soak it in PBS several times after 10 min at room temperature to remove excess dye. The upper chamber fluid was aspirated, and the cells on the surface of the upper chamber membrane were carefully wiped with a wet cotton swab to remove nonmigrating cells. The migrating cells were observed under an inverted microscope and counted (Nikon, Japan) [27].

### 2.6. Identification of EnMT by Immunofluorescence Labeling

Endothelial cell slides were prepared, and the cells were fused to 70% to 80%. Then, the cells were removed, the culture solution was decanted, and the cells were fixed with −20°C prechilled absolute ethanol. TritonX-100 broke the membrane. Add diluted primary antibody, including *α*-SMA (ab5831, Abcam, Cambridge, MA, USA), vWF (ab6994, Abcam, Cambridge, MA, USA). The primary antibodies were all diluted with PBS (1 : 200) and placed in a wet box at 4°C overnight. On the second day, secondary antibodies were added dropwise, incubated at 37°C, nucleus stained with DAPI, antifungal coverslips, and pictures were taken under a fluorescent microscope. [10].

### 2.7. Western Blot Detection of Protein Expression

Total protein was extracted from two large groups (normoxic group and hypoxic group), and the protein concentration was determined by the BCA method [16]. According to western blot procedures and requirements, we prepare 10% separation gel and 5% concentrated gel. The amount of protein loaded in each lane is kept the same. After SDS-PAGE constant pressure electrophoresis, the membrane is transferred and blocked with skim milk. We incubate the VE-cadherin (ab205336, 1 : 1000 dilution; Abcam, Cambridge, MA, USA), Vimentin (ab8978, 1 : 1000 dilution, Abcam, Cambridge, MA, USA), and *ß*-actin (ab8226, 1 : 1000 dilution, Abcam, Cambridge, MA, USA) overnight at 4°C. The secondary antibodies were then incubated at room temperature for 1 hour. Exposure and imaging: the gel image analysis system semiquantifies the absorbance of the obtained band and calculates the ratio of the absorbance of the band to be measured to the internal reference absorbance.

### 2.8. RT-PCR Assays

The total RNA in cells was extracted by Trizol, chloroform/isopropanol method, followed by the reverse transcription kit instructions, and then reverse-transcribed into cDNA for PCR amplification. The primers were designed and synthesized by Shanghai Shenggong Biological Engineering Co., Ltd. The PCR reaction conditions were predenaturation at 94°C for 2 min, denaturation at 94°C for 30 s, extension at 90°C for 90 s after completion of the reaction, and final extension for 2 min. In this study, U6 was used as the internal reference for miRNA detection, and GPADH was used as the internal reference for mRNA detection. Notch3: F: 5′-TGGCGACCTCACTTACGACT-3′, R: 5′-CACTGGCAGTTATAGGTGTTGAC-3'; Jagged-1: F: 5′-GTCCATGCAGAACGTGAACG-3′, R: 5′-GCG GGACTGATACTCCTTGA-3'; U6 : F: 5′-GCTTCGG CAGCACATATACTAAAAT-3′, R: 5′-CGCTTCAGAATTTGCGTGTCAT-3'; GAPDH : F: 5′-CGCTCTCTGCTCCTCCTGTTC-3′, R: 5′-ATCCGTTGACTCCGACCTTCAC-3'. 5 *μ*L of the amplified product was electrophoresed on a 0.5% agarose gel. The gel digital imaging system was used to scan and analyze the amplified product bands, and the absorbance of each amplified band was measured. The absorbance ratio of the amplified band of the target gene to the internal reference *ß*-actin amplified band was used as the relative mRNA expression level index [28].

### 2.9. Dual-Luciferase Reporter Assay

The 3'-UTR sequence of NOTCH3 containing the miR-7-5p binding site was cloned and constructed into a pGL3 vector (E1910, Promega, USA) to generate a wild-type NOTCH3 reporter gene (NOTCH3-Wt). GeneArt™ site-directed mutagenesis system (Thermo Fisher Scientific) was used to generate the mutant NOTCH3 reporter gene (NOTCH3-Mut). NOTCH3-Wt or NOTCH3-Mut and miR-NC and miR-7-5p mimic enter EC cells [15]. 48 hours after transfection, 40 *μ*L stop reagent Renilla luciferase was added after the determination of the firefly luciferase fluorescence value, and the luciferase activity was measured by the luciferase reporter gene assay system (Promega, Madison WI, USA).

### 2.10. miRNA Pull-Down Experiment

The 3'-end biotinylated miR-7 mimics were transfected into the cells. The miRNA pull-down lysis buffer was configured [5]. The lysate consists of 20 mM Tris-HCl (pH = 7.5), 100 mM KCl, 5 nM MgCl2, and 0.3% NP-40. Dynabeads MyOne amyloid magnetic beads are washed with cleavage buffer. We rotate it at 4° for 2 hours. The blocking solution consists of yeast tRNA and BSA. The magnetic beads are washed with a cracking buffer. Magnetic beads were incubated with cell lysate and miRNA. We isolate biotin-labeled miRNAs and their interacting RNAs. QRT-PCR was used to detect the RNA interaction of miRNA.

### 2.11. Statistical Analysis

Statistical analysis was performed using SPSS18.0 software (SPSS Inc., Chicago, IL, USA). Data of triplicate samples from three independent experiments are presented as the mean ± SD. The multicomponent comparison was analyzed using one-way ANOVA. The mean of two samples is tested by *t*-test. *P* < 0.05 was considered statistically significant (^*∗*^p < 0.05; ^*∗∗*^p < 0.01; ^*∗∗∗*^p < 0.001; ns, not significant).

## 3. Results

### 3.1. Hypoxia-Induced Endothelial Cell EnMT

**Figure 1:**
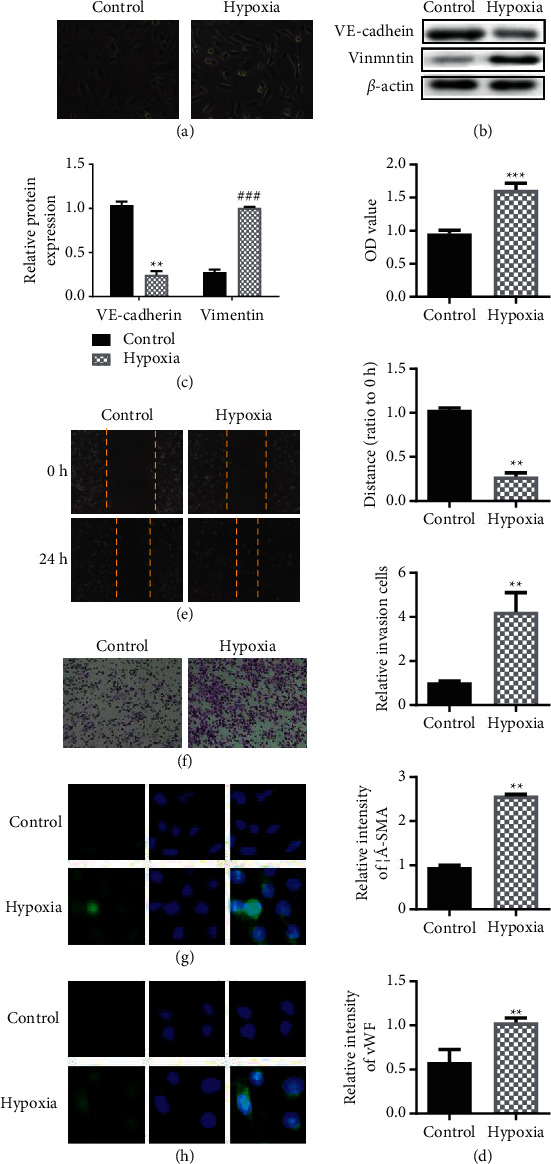
EnMT in endothelial cells induced by hypoxia. (a) Observation of endothelial cell morphology. (b) Detection of the expression of VE-cadherin and Vimentin by western blot experiment. (c) Histogram of protein expression. (d) Detection of the cell viability of endothelial cells by MTT analysis. (e) Transwell assay to evaluate cell migration ability. (f) Scratch test to evaluate cell migration ability. (g) The fluorescent microscope of immunofluorescence staining shows the intensity of *α*-SMA antibody staining. DAPI was used for nuclear staining. (h) The fluorescent microscope of immunofluorescence staining shows vWF expression detection. DAPI was used for nuclear staining. ^*∗∗*^*P* < 0.01, Student's *t*-test. All data are expressed as the mean ± SD of three replicate experiments.

In order to study the effect of hypoxia on pulmonary arterial endothelial cells, we first construct a hypoxia-induced model of pulmonary arterial endothelial cells. Experimental results show that hypoxia can induce endothelial cells to undergo morphological changes. Hypoxia can induce endothelial cells to change from a single-layered cobblestone-like structure to a spindle-like structure, and the cells are arranged disorderly (Figure 1(a)). Compared with the control group, the hypoxia suppressed epithelial marker VE-cadherin expression, and the mesenchymal marker Vimentin expression increased (Figures 1 (b) and 1(c)). Compared with the control group, the cell viability ability of the hypoxia-induced group was significantly enhanced (Figure 1(d)). Further cell scratches and transwell experiments showed that hypoxia induction can promote endothelial cell migration capabilities (Figures 1 (e) and 1(f)). Compared with the blank group, the expression levels of *α*-SMA and vWF in the hypoxia model group showed an upward trend, indicating that hypoxia induced the mesenchymal transition of endothelial cells (Figures 1 (g) and 1(h)).

### 3.2. Hypoxia Induces Upregulation of NOTCH3 Signaling Pathway

**Figure 2:**
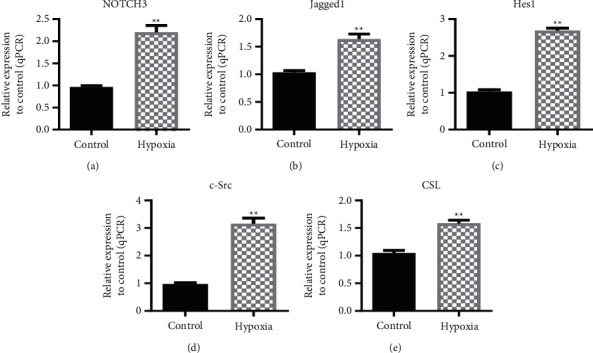
Hypoxia-induced upregulation of the NOTCH3 signaling pathway. (a) RT-qPCR was used to detect the expression of NOTCH3 after hypoxia. (b) RT-qPCR analysis of Jagged-1 expression by hypoxia-induced. (c) RT-qPCR analysis of Hes1 expression by hypoxia-induced. (d) RT-qPCR analysis of c-Src expression by hypoxia-induced. (e) RT-qPCR analysis of CSL expression by hypoxia-induced. ^*∗∗*^*P* < 0.01, Student's *t*-test. All data are presented as the mean ± SD for biological triplicate experiments.

In order to study the molecular mechanism of hypoxia-induced pulmonary mesenchymal transition, we examined changes in the NOTCH3 pathway. The experimental results showed that the expression levels of NOTCH3, Jagged-1, Hes1, c-Src, and CSL in the hypoxia-induced group increased compared with the normoxic control group (Figure 2).

### 3.3. Block NOTCH3 (DAPT Treatment (NOTCH3-Specific Inhibitor)) Inhibits Hypoxia-Induced Endothelial Cell EnMT

**Figure 3:**
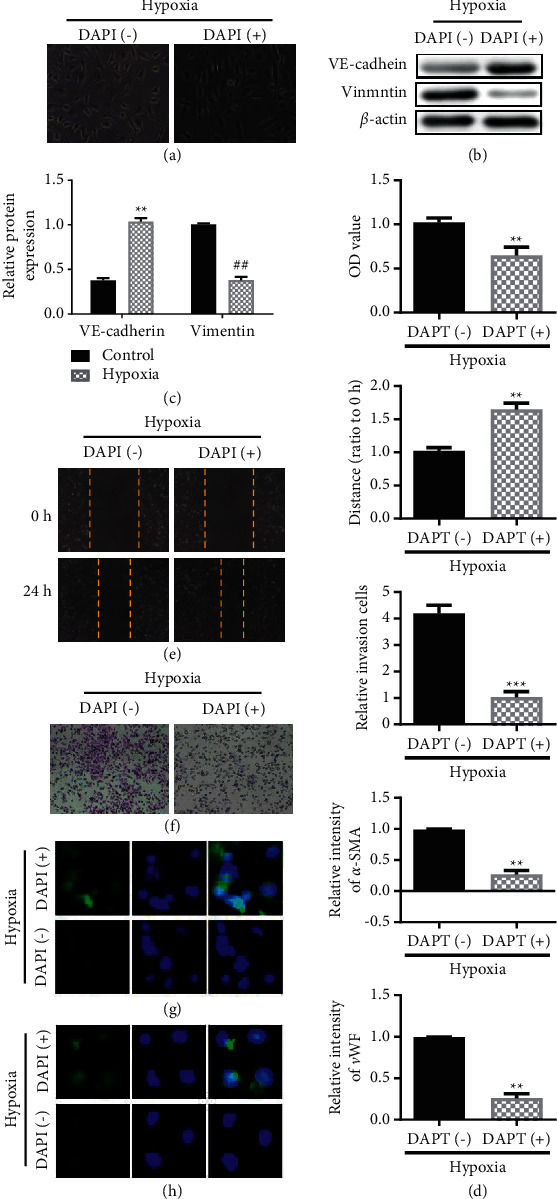
Blocking NOTCH3 (DAPT treatment (NOTCH3-specific inhibitor)) inhibits hypoxia-induced endothelial cell EnMT. (a) Observation of cell morphology. (b) Western blotting analysis of VE-cadherin expression. (c) Western blotting analysis of Vimentin expression. (d) Cell viability experiment on cell proliferation by MTT. (e) Transwell assay to evaluate cell migration. (f) Scratch test to evaluate cell migration. (g) The fluorescent microscope of immunofluorescence staining shows the intensity of *α*-SMA antibody staining. DAPI was used for nuclear staining. (h) The fluorescent microscope of immunofluorescence staining shows the intensity of vWF expression. DAPI was used for nuclear staining. ^*∗∗*^*P* < 0.01, Student's *t*-test. All data are presented as the mean ± SD for biological triplicate experiments.

In order to further verify that hypoxia-induced mesenchymal transition in pulmonary endothelial cells is caused by NOTCH3, we investigated the inhibition of EnMT by inhibiting NOTCH3 through a specific inhibitor of NOTCH3 (DAPT). The experimental results showed that, in the hypoxia-induced background, the endothelial cells did not undergo spindle type changes in the DAPT treatment group added with 50 uMol (Figure 3(a)). Through our previous experimental verification, we found that adding 50 uMol DAPT can inhibit the Notch receptor signaling pathway. In the hypoxia-induced group, the hypoxia-induced epithelial marker VE-cadherin expression decreased, and the mesenchymal marker Vimentin expression increased (Figures 3(b) and 3(c)). At the same time, compared with the hypoxia group, after DAPT inhibited NOTCH3 (Hypoxia + DAPT), the cell viability and migration ability were significantly reduced (Figures 3(d)–3(f)). Furthermore, the expression of *α*-SMA and vWF in endothelial cells in the DAPT-treated group did not increase with hypoxia induction (Figures 3 (g) and 3(h)).

### 3.4. Blocking NOTCH3 (siRNA Jagged-1) Inhibits Hypoxia-Induced Endothelial Cell EnMT

**Figure 4:**
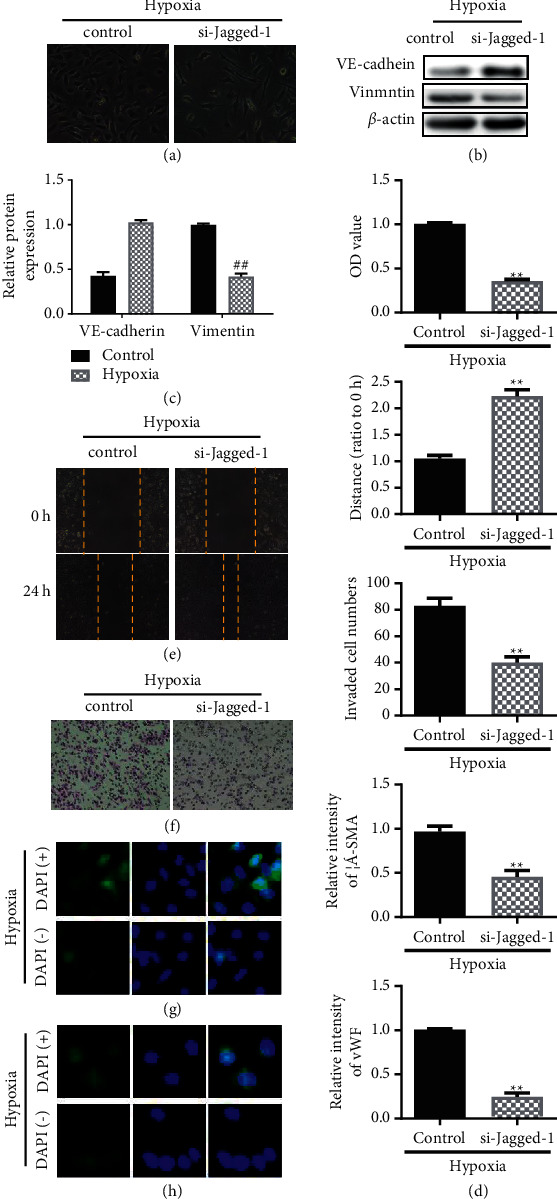
Blocking NOTCH3 (siRNA Jagged-1) inhibits hypoxia-induced endothelial cell EnMT. (a) Observation of cell morphology. (b) Western blotting analysis of VE-cadherin expression. (c) Western blotting analysis of Vimentin expression. (d) Cell viability experiment on cell proliferation by MTT. (e) Transwell assay to evaluate cell migration. (f) Scratch test to evaluate cell migration. (g) The fluorescent microscope of immunofluorescence staining shows the intensity of *α*-SMA antibody staining. DAPI was used for nuclear staining. (h) The fluorescent microscope of immunofluorescence staining shows the intensity of vWF expression. DAPI was used for nuclear staining. ^*∗∗*^*P* < 0.01, Student's *t*-test. All data are presented as the mean ± SD for biological triplicate experiments.

In order to further verify that hypoxia-induced mesenchymal transition of pulmonary endothelial cells is caused by NOTCH3, we knocked down Jagged-1 and studied that blocking the NOTCH3 signaling pathway can inhibit the process of EnMT induced by hypoxia. The experimental results showed that, in the hypoxia-induced background, there was no spindle change in the endothelial cells of the Jagged-1 treatment group (Figure 4(a)). In the hypoxia group, the hypoxia-induced epithelial marker VE-cadherin expression decreased, and the mesenchymal marker Vimentin expression increased (Figures 4(b) and 4(c)). At the same time, compared with the hypoxia group, after Jagged-1 was knocked down, the cell viability and migration ability were significantly reduced (Figures 4(d)–4(f)). Furthermore, the expression of *α*-SMA and vWF in endothelial cells in the Jagged-1 knockdown group did not increase with hypoxia induction (Figures 4(g) and 4(h)).

### 3.5. miR-7-5p Regulates NOTCH3 Expression

**Figure 5:**
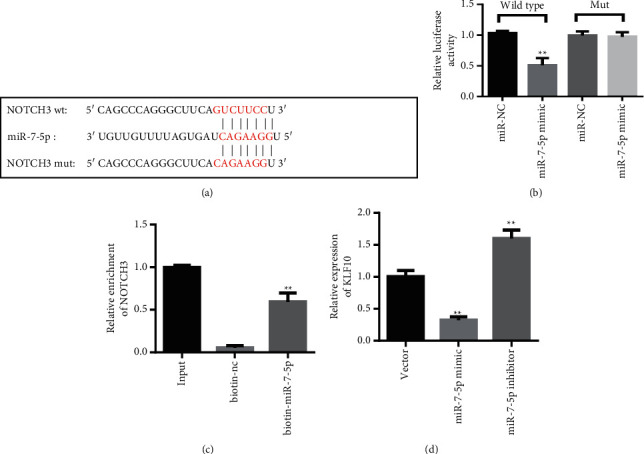
miR-7-5p regulates NOTCH3 expression. (a) Map of miR-7-5p and NOTCH3 binding sites. (b) Double luciferase reporter assay. (c) miRNA pull-down experiment. (d) When miR-7-5p mimics were added to pulmonary artery endothelial cells, NOTCH3 content decreased. With the addition of miR-7-5p inhibitor, NOTCH3 levels increased. ^*∗∗*^*P* < 0.01, one-way ANOVA. All data are presented as the mean ± SD for biological triplicate experiments. All data are presented as the mean ± SD for biological triplicate experiments.

To further study the reasons for the change in the expression of NOTCH3, we analyzed the information of miRNAs that regulate NOTCH3. TargetScan predicts that NOTCH3 can be regulated by miR-7-5p (Figure 5(a)). Further double luciferase reporter assay results confirmed that miR-7-5p can directly bind to and regulate NOTCH3 (Figure 5(b)). Results of miRNA pull-down experiments show that the miR-7-5p probe can catch NOTCH3, further confirming the binding relationship between the two (Figure 5(c)). Further experimental results showed that miR-7-5p mimics added to pulmonary artery endothelial cells reduced the content of NOTCH3. The addition of miR-7-5p inhibitors increased NOTCH3 levels (Figure 5(d)).

### 3.6. miR-7-5p Reduces EnMT in Pulmonary Artery Endothelial Cells by Inhibiting NOTCH3

**Figure 6:**
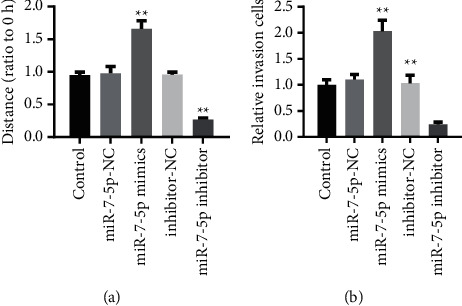
miR-7-5p reduces EnMT in pulmonary arterial endothelial cells by inhibiting NOTCH3. (a) Cell migration experiment. Adding miR-7-5p mimics to the pulmonary artery endothelial cell line reduced cell migration capacity. Adding miR-7-5p inhibitors enhances cell migration. (b) Cell migration experiments. Adding miR-7-5p mimics to the pulmonary artery endothelial cell line reduced cell migration capacity. Adding miR-7-5p inhibitors enhances cell migration ability. ^*∗∗*^*P* < 0.01, one-way ANOVA. All data are presented as the mean ± SD for biological triplicate experiments.

Adding miR-7-5p mimics to the pulmonary artery endothelial cell line reduced cell migration capacity. Adding miR-7-5p inhibitors increased cell migration (Figure 6(a)). Adding miR-7-5p mimics to the pulmonary artery endothelial cell line reduced cell migration capacity. Adding miR-7-5p inhibitors enhanced cell migration capacity (Figure 6(b)).

## 4. Discussion

In this study, PAECs were cultured in vitro, endothelial cell morphology was observed under an inverted phase-contrast microscope, and endothelial-mesenchymal transition experiments were performed on pulmonary artery endothelial cells through cell viability experiments, cell scratch experiments, and cell transwell experiments.

Chronic hypoxia participates in various pathological processes in the body. In the rat unilateral ureteral obstruction (UUO) renal fibrosis model, hypoxia is considered to be one of the factors promoting fibrosis [29], and its key mechanism is to promote endothelial cells. Phenotypic transformation occurs and becomes an important source of activating fibroblasts in fibrotic lesions. In addition, in the hypoxic environment, the endocrine function of endothelial cells is disturbed, and pathogenic factors are activated, leading to disease. In this experiment, PAECs were cultured under hypoxia and normoxic for a certain period of time by simulating the hypoxic environment in the body. The results showed that the morphology of endothelial cells in the hypoxia group was significantly changed compared with endothelial cells in the normoxic group. The cobblestone-like structure changed into a long spindle-like structure, and the cells were arranged randomly. Western blot and RT-PCR results confirmed that hypoxia promoted expression of endothelial cell marker VE-cad decreased and the expression of mesenchymal marker Vimentin increased. Immunofluorescence experiments further confirmed that *α*-SMA expression was not found in cells cultured in normoxic culture, but *α*-SMA was expressed in endothelial cells cultured in hypoxia. Those results indicated that endothelial cells had undergone an interstitial transformation.

The transformed cells may form activated fibroblasts, which are involved in the occurrence of pulmonary fibrosis and tumor microenvironment formation [30]. Hypoxia can significantly enhance the migration of endothelial cells and cause them to migrate to the media layer. After EnMT, the median thickness of the hypoxic pulmonary artery can be increased, and the pulmonary artery can be narrowed, leading to the occurrence of pulmonary hypertension and chronic obstructive pulmonary disease. Use of NOTCH3-specific inhibitor DAPT and knockdown of Jagged-1 can inhibit the induction of EnMT by hypoxia, indicating that NOTCH3 has an important role in EnMT.

This study also found that miR-7-5p can regulate NOTCH3 in endothelial cells. MicroRNA-7 (miR-7) is a multifunctional miRNA that plays multiple roles in physiological and pathological conditions. Studies have shown that miR-7-5p has a tumor suppressor effect in gastric cancer, colorectal cancer, and glioblastoma [31]. Recent studies have found that miR-7-5p also plays a vital role in tumor metastasis [32]. The binding sites of miR-7-5p and NOTCH3 were predicted and analyzed by TargetScan. Furthermore, the regulation effect of miR-7-5p on NOTCH3 was further confirmed by a double luciferase reporter assay and miRNA pull-down experiment. Those results indicated that miRNAs are also involved in the process of endothelial cell mesenchymal transition.

## 5. Conclusion

**Figure 7:**
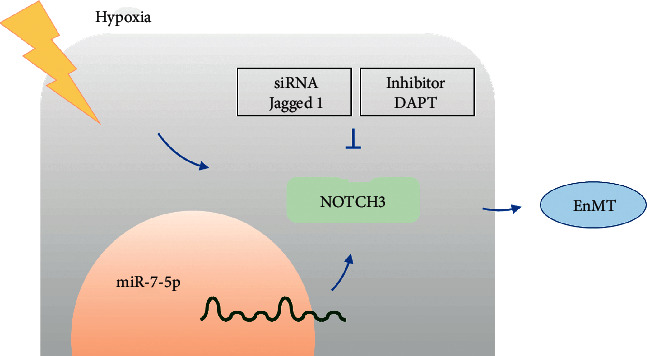
Graphical abstract. Hypoxia upregulates the NOTCH3 signaling pathway to promote endothelial-mesenchymal transition in pulmonary artery endothelial cells.

In this study, we found that hypoxia upregulates the NOTCH3 signaling pathway to promote EnMT in pulmonary artery endothelial cells. And our research found that miR-7-5p can reduce EnMT in pulmonary artery endothelial cells by inhibiting NOTCH3 (Figure 7). Therefore, NOTCH3 can become a breakthrough in the study of EnMT in endothelial cells to study more EnMT-related diseases. This study enriches the understanding of the mechanism of EnMT regulating disease. A more comprehensive and in-depth understanding of the process of endothelial-mesenchymal transition is needed in the future. It is expected to help provide more effective diagnosis and treatment measures for solving various diseases.

## Data Availability

The authors can make data available on request through a data access committee and institutional review board. In addition, all the data can also be obtained from author Li-Le Wang.
